# Addressing factors associated with nursing workforce instability: the mediating effect of professional identification between perceived responses to capitalization attempts and job burnout in newly recruited nurses

**DOI:** 10.3389/fpubh.2026.1784834

**Published:** 2026-03-16

**Authors:** Fuhai Xia, Gen Li, Liqin Xu, Li Li, Xi Chen, Qiang Li

**Affiliations:** Department of Operating Room, The Central Hospital of Wuhan, Tongji Medical College, Huazhong University of Science and Technology, Wuhan, China

**Keywords:** mediation analysis, nurses, professional burnout, professional identity, social support

## Abstract

**Introduction:**

Newly recruited nurses are prone to negative emotions and have a high incidence of job burnout due to insufficient work experience and weak ability to handle clinical emergencies. Different modes of perceived responses to capitalization attempts may affect their job burnout through professional identification, and the specific mechanism of action among the three has not been clarified yet.

**Objectives:**

To explore the mediating role of professional identification between perceived responses to capitalization attempts and job burnout among newly recruited nurses, and to provide theoretical basis and practical reference for burnout intervention in this group.

**Methods:**

A cross-sectional study was conducted. From May to August 2024, 232 newly recruited nurses ( ≤ 2 years of clinical experience) from two tertiary hospitals in Wuhan were selected via cluster sampling. Data were collected using the Perceived Responses to Capitalization Attempts Scale, Professional Identification Scale, and Chinese version of Maslach Burnout Inventory. Pearson correlation, multiple linear regression, and structural equation modeling with bias-corrected bootstrapping were used for analysis.

**Results:**

Job burnout was negatively correlated with active-constructive (*p* < 0.05) and professional identification (*p* < 0.01), but positively correlated with passive-constructive, active-destructive and passive-destructive (*p* < 0.01). Professional identification was positively correlated with active-constructive (*p* < 0.01), and negatively correlated with passive-constructive (*p* < 0.05), active-destructive (*p* < 0.05) and passive-destructive (*p* < 0.01). The results of multiple linear regression analysis showed that passive-constructive (*p* = 0.03) and passive-destructive (*p* < 0.001) could positively predict job burnout, while professional identification could negatively predict job burnout (*p* < 0.001). The structural equation model showed that professional identification exhibited a complete mediating effect (72.22%) between passive-constructive and job burnout. Professional identification exhibited a partial mediating effect (59.46%) between passive-destructive and job burnout.

**Conclusion:**

This study reveals, within the Job Demands-Resources and positive psychology frameworks, the mediating role of professional identification in how perceived responses to capitalization attempts affect job burnout in new nurses. The findings offer empirical support for developing targeted strategies to enhance professional identity, reduce burnout, and address factors associated with nursing workforce instability.

## Introduction

Globally, nursing workforce shortages have emerged as a core challenge for healthcare systems, with high rates of job burnout and turnover among newly recruited nurses (those with ≤ 2 years of clinical nursing experience after graduation) being key contributors to this crisis (1). In the early stages of their careers, nurses often face role transition shocks and work pressure due to insufficient clinical experience and weak emergency response capabilities, which easily trigger negative emotions and further lead to job burnout—a phenomenon that is prominent worldwide (2). According to a cross-national survey by Cañadas-De la Fuente et al., the prevalence of burnout syndrome among nurses globally ranges from 30 to 70%, with the incidence among nurses within 2 years of employment significantly higher than that among senior nurses (3). In some countries, the turnover rate of this group even exceeds 30%, directly threatening the stability of the nursing workforce and the quality of medical services (3). A longitudinal study in the Canada showed that the incidence of job burnout among newly recruited nurses in their first year of practice reached 48.9%, and burnout levels were significantly positively correlated with turnover intention (4). Research in Japan has also confirmed that emotional exhaustion caused by poor occupational adaptation among new nurses reduces the quality of their nursing services and increases the risk of adverse medical events (5). In China, the problem of job burnout among newly recruited nurses is equally severe, with relevant data showing that the incidence of job burnout ranges from 45.4 to 76.9%, and job burnout significantly increases their turnover intention, further exacerbating the domestic nursing workforce shortage (6).

Professional identity, defined as nurses' positive cognition and emotional connection to their profession, is a core psychological resource for alleviating job burnout and maintaining occupational stability (7). From the perspective of the Job Demands-Resources (JD-R) Model (8), professional identity is a key personal resource that can buffer the pressure caused by job demands (e.g., heavy workloads, emergency tasks) and reduce the risk of job burnout. A study by Zborowska et al. confirmed that the stronger nurses' professional identity, the lower their level of job burnout, and professional identity can enhance nurses' occupational resilience by improving their sense of work meaning (9). In the fields of nursing education and management, strengthening new nurses' professional identity has become a core measure to improve their occupational adaptation. An intervention study by Hu et al. showed that targeted occupational transition training can significantly improve the professional identity of new nurses in the intensive care unit and reduce their turnover intention (10).

From the perspective of positive psychology, individuals' sharing of positive events and corresponding feedback (i.e., perceived responses to capitalization attempts) is an important way to stimulate positive emotions and build social support (11). This concept includes four response modes: active-constructive (enthusiastic feedback), passive-constructive (plain recognition), active-destructive (negative denial), and passive-destructive (indifference and neglect). According to the JD-R Model, positive responses to capitalization attempts can be regarded as supplements to work resources, which can enhance individuals' psychological capital; while negative responses will exacerbate the pressure brought by job demands and weaken occupational adaptability (12, 13). Existing studies have confirmed that perceived responses to capitalization attempts are closely related to individuals' subjective wellbeing (14), work engagement (15), and professional identity (16), and will also affect the quality of workplace interpersonal relationships (17). For example, a survey by Chen et al. on nurses in Grade A tertiary hospitals in Hangzhou found that active-constructive responses to capitalization attempts can significantly improve nurses' work immersion (15); a study by Jiang et al. also pointed out that insufficient capitalization support will exacerbate job burnout among ICU nurses (16).

Although independent studies on job burnout, professional identity, and perceived responses to capitalization attempts among newly recruited nurses are abundant, the mechanism of interaction among the three variables has not been systematically explored within the same framework. There are two key gaps in existing research: first, there is a lack of research on the impact of perceived responses to capitalization attempts on job burnout among newly recruited nurses, which hinders the exploration of factors associated with nursing workforce instability; second, the mediating path of professional identity between perceived responses to capitalization attempts and job burnout has not been clarified by combining the JD-R Model and positive psychology theories, which cannot provide an intervention framework with both theoretical support and practical operability for nursing management. Against the background of intensifying global competition for nursing human resources and positive psychological intervention becoming a mainstream strategy for occupational adaptation, the integrated perspective of the three variables in this study not only fills the theoretical gap in the field of nursing science but also provides references for China to formulate intervention programs for new nurses' occupational adaptation, which has important practical and academic contributions to addressing factors associated with nursing workforce instability.

Based on the above literature review, two hypotheses are proposed:

1. ***H1*** Perceived responses to capitalization attempts will predict job burnout.
2. ***H2*** Professional identification will mediate the relationship between perceived responses to capitalization attempts (four types) and job burnout.

## Materials and methods

### Study design

This study is a cross-sectional study and follows STROBE statement. From May–August 2024, cluster sampling was employed to recruit 232 newly recruited nurses from two tertiary hospitals in Wuhan. Specifically, the “clusters” were defined as clinical departments (e.g., internal medicine, surgery, emergency department, intensive care unit) within the two hospitals. First, 6 clinical departments were randomly selected from each hospital using a random number table method; then, all newly recruited nurses who met the inclusion criteria in the selected departments were invited to participate in the study, ultimately enrolling 232 eligible subjects. Newly recruited nurses refer to nurses who have been engaged in clinical nursing for less than 2 years after graduation (1).

Sampling criteria for clinical departments: (1) The total number of clinical departments in the two research hospitals (38 and 42 respectively, 80 in total); (2) Department inclusion criteria: clinical departments with ≥5 newly recruited nurses, excluding medical technology departments and administrative departments; (3) Sampling and participation: 12 departments (6 per hospital) were randomly selected from 80 departments using a random number table method, all selected departments agreed to participate with no refusals, and all newly recruited nurses in the finally included departments met the inclusion criteria of the study subjects.

Inclusion criteria of the study subjects: (1) registered nurses; (2) have completed pre-job training and engaged in clinical nursing work; (3) informed consent and voluntary participation in this study; (4) experience less than 2 years. Exclusion criteria of the study subjects: (1) absent for more than 1 month. For mediation effect analysis, the sample size should reach 10–15 times the number of observed variables. Sample size estimation is based on 10–20 times the number of independent variables (passive-constructive with 3 items, passive-destructive with 3 items, professional identification with 3 dimensions, job burnout with 3 dimensions, and 6 sociodemographic variables). This study includes 18 independent variables and accounts for a 10% nonresponse rate, requiring a sample size ranging from 198–396 participants (18, 19). The actual recruited sample size of 232 participants meets the requirement. Of the 251 participants who participated in the study, 19 declined to participate and 232 accepted, a response rate of 92.4%.

### Measures

Sample characteristics: including gender, age, education, hire date, labor relation and the level of the trainee hospital.

Chinese version of Maslach burnout inventory (CMBI). This scale was compiled by Zhu et al. (20), mainly used for assess an individual's levels of Job burnout. The CMBI includes three dimensions: emotional exhaustion, depersonalization and personal accomplishment. A six-point Likert scale (0 = “never” −6 = “often”) is used and the total score of 15 items varies from 0 to 90, with higher scores suggesting a higher levels of Job burnout. During the scale development phase, the Cronbach's α coefficients for the total score and each dimension ranged from 0.67 to 0.87. In this study, the Cronbach's α coefficients for the total score and each dimension ranged from 0.85 to 0.93.

Perceived responses to capitalization attempts scale (PRCA). The PRCA is used to assess an individual's response to positive events (11). The PRCA includes four types: active-constructive, passive-constructive, active-destructive and passive-destructive. A seven-point Likert scale (1 = “completely inconsistent” to 7 = “completely consistent”) is used and the total score of 12 items varies from 3 to 21. The higher the score of a type, the more dominant the response mode represented by that type compared to the other types. During the scale development phase, the Cronbach's α coefficients for the total score and each dimension ranged from 0.88 to 0.91. In this study, the Cronbach's α coefficients for the total score and each dimension ranged from 0.75 to 0.82.

Professional identification scale (PIS). The PIS is used to assess an individual's levels of professional identification (21). The PIS includes three dimensions: awareness, evaluation and affect. A five-point Likert scale (1 = “never” to 5 = “often”) is used and the total score of 10 items varies from 10 to 50, with higher scores suggesting a higher levels of professional identification. During the scale development phase, the Cronbach's α coefficient for the total scale score was 0.71; in this study, the Cronbach's α coefficients for the total score and each dimension ranged from 0.83 to 0.92.

### Data collection

After obtaining the consent of the director of the nursing department of the hospital, newly recruited nurses were invited to fill in the electronic questionnaire through WeChat. All participants gave their voluntary written informed consent prior to study participation. There were no missing values in the 232 questionnaires.

### Data analysis

SPSS version 26.0 and AMOS version 24.0 were used for statistical analysis. The Shapiro-Wilk test confirmed the normality of the data (*p* > 0.05). The data in this study conform to a normal distribution. Mean and standard deviation (SD) were used to describe continuous variables. Pearson correlation analysis was used to analyze the relationships between the study variables. Exploratory factor analysis was used to test common method biases. Confirmatory factor analysis was employed to calculate item factor loadings, composite reliability (CR), and average variance extracted (AVE). Multiple linear regression was used to analyze the influencing factors of job burnout. Validate the model for multicollinearity issues using the Durbin-Watson (D-W) statistic and the variance inflation factor (VIF). Structural equation model was used to construct and evaluate the mediation model. Bias-corrected bootstrapping method was used to test the significance of the mediating effect. All statistical tests were conducted by two-sided tests, and *p* values of < 0.05 indicated statistical significance.

### Ethics approval and consent to participate

The studies involving humans were approved by The Central Hospital of Wuhan ethics committee. The studies were conducted in accordance with the local legislation and institutional requirements. The participants provided their written informed consent to participate in this study. The social media data was accessed and analyzed in accordance with the platform's terms of use and all relevant institutional/national regulations.

## Results

### Sample characteristics

Of the 232 participants, 195 were female, accounting for 84.05%. The average age was 23.26 years (*SD* = 1.19; range from 21 to 28), 202 were educated more than undergraduate, accounting for 87.69%; 228 had interns in tertiary hospitals. There were 221 participants whose labor relations were based on contracts. The average hire date was 12.09 months (*SD* = 5.87; range from 6 to 23).

### Job burnout, professional identification and perceived responses to capitalization attempts scores

The results of this study showed that the mean score of job burnout was 29.91 (*SD* = 16.42). The mean score of professional identification was 40.28 (*SD* = 7.35). The mean score of the four types of the perceived responses to capitalization attempts were: active-constructive 14.65 (*SD* = 3.53), passive-constructive 13.96 (*SD* = 4.12), active-destructive 13.74 (*SD* = 3.77) and passive-destructive 10.51 (*SD* = 3.72). More detailed scoring information is reported in Table 1.

**Table 1:** The scores of all variables.

**Variable**	**Mean ±SD**
Active-constructive	14.65 ± 3.53
Passive-constructive	13.96 ± 4.12
Active-destructive	13.74 ± 3.77
Passive-destructive	10.51 ± 3.72
Professional identification	40.28 ± 7.35
Awareness	11.59 ± 2.76
Evaluation	12.44 ± 2.43
Affect	16.26 ± 3.36
Job burnout	29.91 ± 16.42
Emotional exhaustion	14.72 ± 7.31
Depersonalization	7.77 ± 6.00
Personal accomplishment	7.42 ± 7.61

### Correlations of job burnout, professional identification and perceived responses to capitalization attempts

The results showed that job burnout was negatively correlated with active-constructive (*r* = −0.17, *p* < 0.05) and professional identification (*r* = −0.77, *p* < 0.01), but positively correlated with passive-constructive, active-destructive and passive-destructive (*r* = 0.23, 0.17, 0.43, *p* < 0.01). Professional identification was positively correlated with active-constructive (*r* = 0.21, *p* < 0.01), and negatively correlated with passive-constructive (*r* = −0.16, *p* < 0.05), active-destructive (*r* = −0.13, *p* < 0.05) and passive-destructive (*r* = −0.28, *p* < 0.01). Table 2 shows the correlations of job burnout, professional identification and perceived responses to capitalization attempts.

**Table 2:** Correlations of job burnout, professional identification and perceived responses to capitalization attempts.

**Variable**	**1**	**2**	**3**	**4**	**5**	**6**	**7**	**8**	**9**	**10**	**11**	**12**
1. Active-constructive	1.00											
2. Passive-constructive	0.14^*^	1.00										
3. Active-destructive	0.30^**^	0.36^**^	1.00									
4. Passive-destructive	0.07	0.14^*^	0.20^**^	1.00								
5. Professional identification	0.21^**^	−0.16^*^	−0.13^*^	−0.28^**^	1.00							
6. Awareness	0.26^**^	−0.08	−0.12	−0.06	0.82^**^	1.00						
7. Evaluation	0.05	−0.15^*^	−0.15^*^	−0.39^**^	0.81^**^	0.43^**^	1.00					
8. Affect	0.21^**^	−0.17^**^	−0.08	−0.28^**^	0.93^**^	0.66^**^	0.70^**^	1.00				
9. Job burnout	−0.17^*^	0.23^**^	0.17^**^	0.43^**^	−0.77^**^	−0.58^**^	−0.64^**^	−0.75^**^	1.00			
10. Emotional exhaustion	−0.16^*^	0.19^**^	0.18^**^	0.35^**^	−0.55^**^	−0.44^**^	−0.45^**^	−0.51^**^	0.83^**^	1.00		
11. Depersonalization	−0.16^*^	0.16^*^	0.13^*^	0.33^**^	−0.64^**^	−0.49^**^	−0.52^**^	−0.63^**^	0.88^**^	0.80^**^	1.00	
12. Personal accomplishment	−0.07	0.19^**^	0.09	0.34^**^	−0.63^**^	−0.44^**^	−0.53^**^	−0.64^**^	0.67^**^	0.19^**^	0.34^**^	1.00

^*^P < 0.05.

^**^P < 0.01.

### Multiple linear regression analysis of job burnout in newly recruited nurses

We designed a multiple linear regression model with job burnout as the dependent variable, the four dimensions of perceived responses to capitalization attempts and job identification as the independent variables. The results of multiple linear regression analysis showed that passive-constructive (*B* = 0.37, 95% CI [0.04, 0.70], *p* = 0.03) and passive-destructive (*B* = 1.01, 95% CI [0.65, 1.37], *p* < 0.001) could positively predict job burnout, while professional identification could negatively predict job burnout (*B* = −1.51, 95% CI [−1.70, −1.32], *p* < 0.001). In the regression equation, *R*^2^ = 0.66, Δ*R*^2^ = 0.65, *F* = 85.72, *p* < 0.001. The results of multiple linear regression analysis indicate that the D-W value is 1.82, and all VIF values are less than 2. Therefore, no severe multicollinearity issues exist in the linear regression model.

### Measurement model validation

The measurement model was evaluated for convergent validity and discriminant validity to ensure the robustness of the constructs.

The analysis of convergent validity revealed that all dimensions demonstrated satisfactory internal consistency and reliability. Specifically, the Cronbach's α coefficients for the dimensions ranged from 0.75 to 0.89, indicating good internal consistency across the scales. The CR values were between 0.79 and 0.91, all exceeding the threshold of 0.7, which confirms excellent aggregation of items under each dimension. Additionally, the AVE values varied from 0.53 to 0.66, meeting the criterion of ≥0.5. This suggests that the dimensions effectively captured a substantial portion of the variance from their respective items, supporting adequate convergent validity. Table 3 presents the convergent validity of the scale dimensions.

**Table 3:** Convergent validity of scale dimensions.

**Scales**	**Dimensions**	**Entries**	**Cronbach's α**	**CR**	**AVE**
PRCA	Active-constructive	3	0.78	0.82	0.56
	Passive-constructive	3	0.77	0.80	0.55
	Active-destructive	3	0.75	0.79	0.53
	Passive-destructive	3	0.78	0.81	0.56
PIS	Awareness	3	0.83	0.86	0.63
	Evaluation	3	0.83	0.86	0.65
	Affect	4	0.86	0.88	0.62
CMBI	Emotional exhaustion	5	0.89	0.91	0.62
	Depersonalization	4	0.85	0.87	0.59
	Personal accomplishment	6	0.88	0.89	0.59

CR, composite reliability; AVE, average variance extraction.

Discriminant validity was assessed by comparing the square roots of the AVE for each dimension with the correlations between dimensions. The square roots of the AVE (diagonal values) ranged from 0.73 to 0.80, and these values were consistently greater than the absolute correlations between dimensions, which ranged from −0.49 to 0.36. Furthermore, the inter-dimensional correlation coefficients did not exhibit extremely high values (all below 0.7), indicating clear conceptual boundaries between constructs and the absence of severe overlap. Thus, the discriminant validity of the measurement model is well-supported. Table 4 presents the correlation coefficient matrix between the square roots of AVE dimensions and scale dimensions.

**Table 4:** Matrix of correlation coefficients between dimensions of AVE square roots and scale dimensions.

**Dimensions**	**1**	**2**	**3**	**4**	**5**	**6**	**7**	**8**	**9**	**10**
1. Active-constructive	0.75									
2. Passive-constructive	0.14^*^	0.74								
3. Active-destructive	0.30^**^	0.36^**^	0.73							
4. Passive-destructive	0.07	0.14^*^	0.20^**^	0.75						
5. Awareness	0.26^**^	−0.08	−0.12	−0.06	0.79					
6. Evaluation	0.05	−0.15^*^	−0.15^*^	−0.39^**^	0.43^**^	0.80				
7. Affect	0.21^**^	−0.17^**^	−0.08	−0.28^**^	0.66^**^	0.70^**^	0.79			
8. Emotional exhaustion	−0.16^*^	0.19^**^	0.18^**^	0.35^**^	−0.44^**^	−0.45^**^	−0.51^**^	0.79		
9. Depersonalization	−0.16^*^	0.16^*^	0.13^*^	0.33^**^	−0.49^**^	−0.52^**^	−0.63^**^	0.80^**^	0.77	
10. Personal accomplishment	−0.07	0.19^**^	0.09	0.34^**^	−0.44^**^	−0.53^**^	−0.64^**^	0.19^**^	0.34^**^	0.77

Diagonal values represent the square root of the AVE for each dimension.

Below the diagonal are the Pearson correlation coefficients between dimensions.

^*^p < 0.05.

^**^p < 0.01.

### Test of common method variance biases

Test of common method variance bias is required when using self-reported data. Such bias must be carefully considered in interpreting study data. There were 7 variables with characteristic roots greater than 1. The first factor could only explain 27.42% of the key standards, less than 40% (22, 23), indicating that there were no severe common method biases in this study.

### Mediation effect analysis

**Figure 1:**
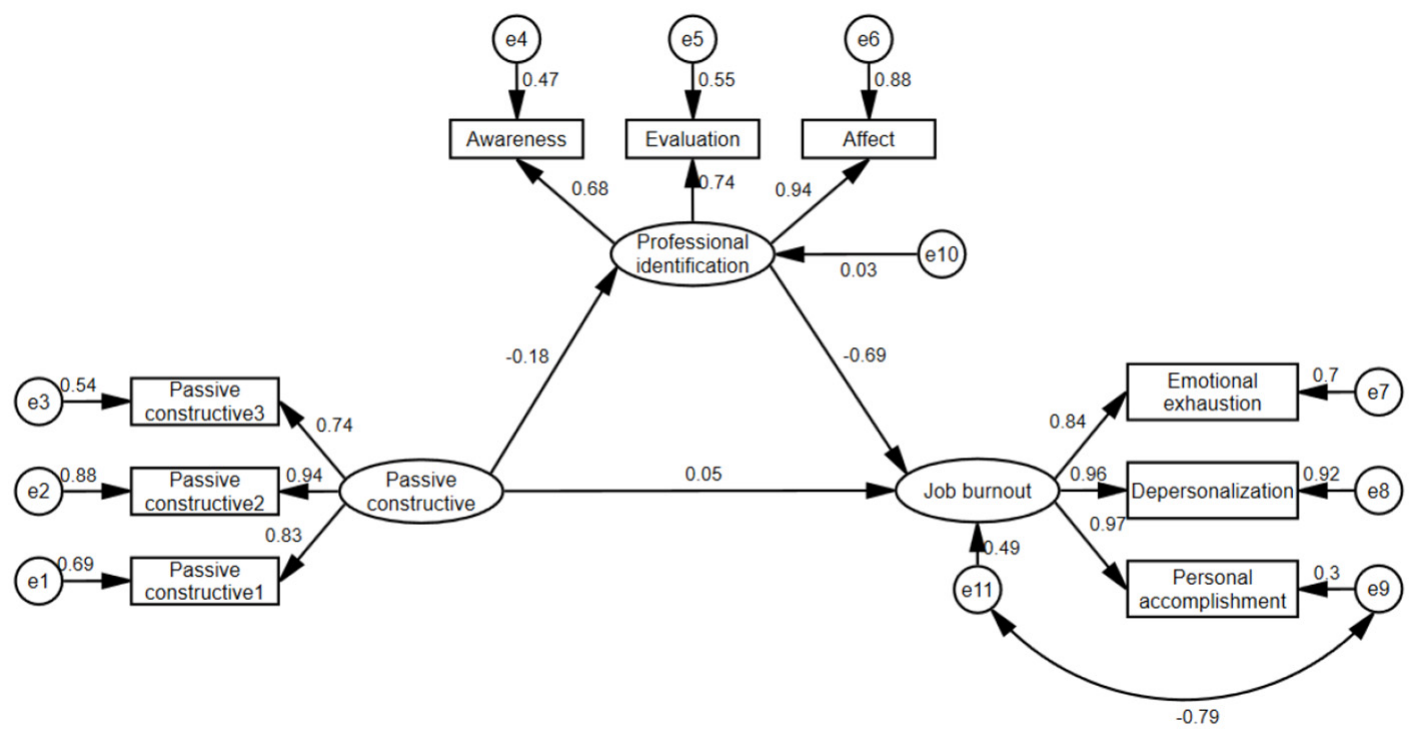
Mediating effect of professional identification between passive-constructive and job burnout in newly recruited nurses (standardization coefficient/β).

**Figure 2:**
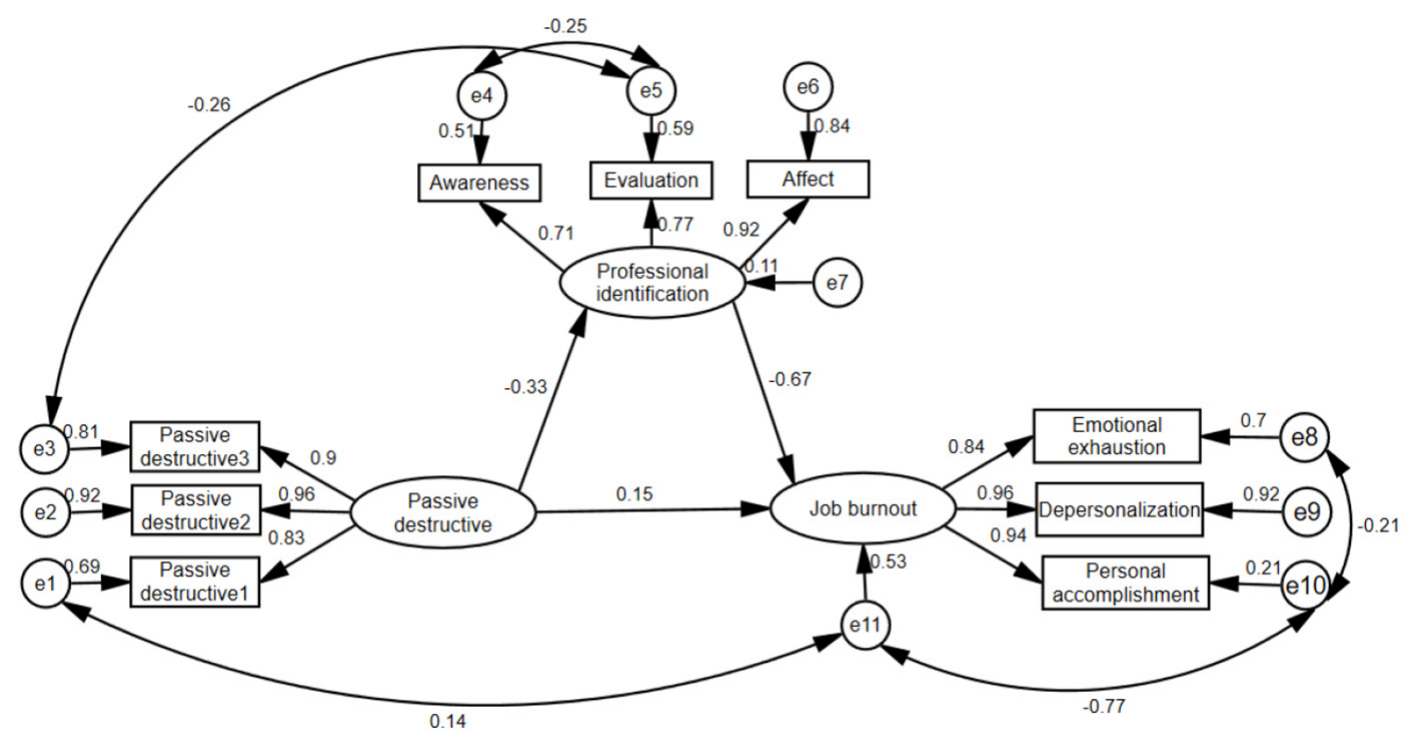
Mediating effect of professional identification between passive-destructive and job burnout in newly recruited nurses (standardization coefficient/β).

Structural equation models were established with passive-constructive and passive-destructive as independent variables, job identification as mediating variables, and job burnout as dependent variables, according to the results of correlation analysis and multiple linear regression analysis. The model fitting results show that the initial model fits poorly. Therefore, the maximum likelihood method and the modified index are used to modify the model, and the modified models 1 and 2 conforming to the standard are finally obtained. Table 5 shows the goodness-of-fit results. Figure 1 shows the mediating effect of professional identification between passive-constructive and job burnout in newly recruited nurses. Figure 2 shows the mediating effect of professional identification between passive-destructive and job burnout in newly recruited nurses.

**Table 5:** The goodness-of-fit results.

**Categories**	***x*^2^/df**	**RMSEA**	**GFI**	**AGFI**	**CFI**
Standard	< 3.00	< 0.08	>0.90	>0.90	>0.90
Initial model 1	6.13	0.15	0.89	0.80	0.89
Modified model 1	1.99	0.06	0.96	0.92	0.98
Initial model 2	7.94	0.17	0.86	0.74	0.86
Modified model 2	2.46	0.07	0.96	0.91	0.98

χ2/df, ratio of chi-square to degrees of freedom; RMSEA, root mean square error of approximation; GFI, goodness-of-fit index; AGFI, adjusted goodness-of-fit index; CFI, comparative fit index.

Bootstrapping with 5,000 repeated samples was employed to test the significance of the mediating effects. A 95% confidence interval (CI) was used, with effects considered significant if the CI did not include zero (24). The results indicated that for Mediation Model 1, the total effect was significant (β = 0.18, 95% CI [0.05, 0.30], *p* < 0.001). The direct effect was not significant (β = 0.05, 95% CI [−0.01, 0.12], *p* = 0.163), whereas the indirect effect was significant (β = 0.13, 95% CI [0.02, 0.23], *p* < 0.001), accounting for 72.22% of the total effect. For Mediation Model 2, the total effect (β = 0.37, 95% CI [0.28, 0.47], *p* < 0.001), direct effect (β = 0.15, 95% CI [0.06, 0.23], *p* < 0.001), and indirect effect (β = 0.22, 95% CI [0.13, 0.32], *p* < 0.001) were all significant. The indirect effect accounted for 59.46% of the total effect. Full mediation means the independent variable influences the dependent variable entirely through the mediator (the direct effect becomes non-significant after controlling for the mediator), while partial mediation means the independent variable influences the dependent variable partially through the mediator and partially directly (the direct effect remains significant after controlling for the mediator). Complete results of the mediation analysis are presented in Table 6.

**Table 6:** Test results of mediating effect.

**Structural paths**	**Standard coefficients (Effect value/β)**	**Effect size**	**95% CI**	***P***
**Mediation model 1**				
Total effect	0.18	100.00%	0.05–0.30	< 0.001
Direct effect	0.05	27.78%	−0.01–0.12	0.163
Indirect effect	0.13	72.22%	0.02–0.23	< 0.001
**Mediation model 2**				
Total effect	0.37	100.00%	0.28–0.47	< 0.001
Direct effect	0.15	40.54%	0.06–0.23	< 0.001
Indirect effect	0.22	59.46%	0.13–0.32	< 0.001

Indirect effect of mediation model 1: passive-constructive → professional identification → job burnout; Indirect effect of mediation model 2: passive-destructive → professional identification → job burnout.

## Discussion

This study is the first to reveal, in the context of addressing factors associated with nursing workforce instability, the mediating role of professional identity between perceived responses to capitalization attempts and job burnout among newly recruited nurses, filling the gap in theoretical research in this field.

### Current status of job burnout

From an international perspective, the high incidence of job burnout among newly recruited nurses has become a global consensus. A cross-national survey by Cañadas-De la Fuente et al. showed that 30%−70% of nurses experience job burnout, with nurses within 2 years of employment facing a significantly higher risk of burnout than senior nurses (3). A longitudinal study in the Canada also confirmed that the incidence of job burnout among new nurses in their first year of practice reached 48.9%, which was highly correlated with turnover intention (4). This is consistent with the baseline characteristics of job burnout among new nurses in Wuhan in this study (total score 29.91 ± 16.42), confirming the global nature of job burnout among new nurses. Among the three dimensions of job burnout, the score of emotional exhaustion is the highest, which may be related to heavy workload, long-term night work, less family support and transition shock (25).

### Current status of perceived responses to capitalization attempts

The scores of the newly recruited nurses in the four dimensions of perceived responses to capitalization attempts were ranked from highest to lowest as active-constructive, passive - constructive, active-destructive, and passive - destructive, which was consistent with the results of a previous study (26). Active-constructive is the enthusiastic and proactive response of the responder to the good things shared by the sharer (26). The reasons why participants in this study chose more active-constructive response are as follows. The clinical department has a standardized, systematic and perfect training program for newly recruited nurses, and senior nurses can respond warmly to questions raised by newly recruited nurses. On the one hand, this kind of support from senior nurses will make newly recruited nurses feel safe, help them identify problems and make good suggestions, and ultimately improve their positive behavior. On the other hand, the enthusiastic responses of the respondents also encouraged the newly recruited nurses to pay more attention to positive events and thus have positive emotions, which could improve their work efficiency and promote the sense of career gain and professional identification.

### Current status of professional identification

The professional identification of newly recruited nurses was at a medium level in this study (15). During the novel coronavirus pandemic, nurses have made important contributions to the health of all humanity, which reflects the value and importance of their profession. However, when new recruited nurses are faced with complex clinical work, they have insufficient experience, poor ability to deal with emergencies, and are prone to emotional exhaustion, which reduces their professional identification (15). The score of evaluation was the highest in all dimensions of professional identification. In recent years, nursing managers in China have paid more attention to the integrated development of newly recruited nurses, adding career planning, humanistic care, book report, film appreciation, academic discussion and other projects in standardized training, which improves nurses' participation in their professional roles and influences their attitude and views on nursing profession. At the same time, it can promote stronger organizational commitment (27).

### Correlation analysis of job burnout, professional identification and perceived responses to capitalization attempts

The results of this study show that job burnout is negatively correlated with active-constructive and professional identification, but positively correlated with passive-constructive, active-destructive and passive-destructive. Professional identification is positively correlated with active construction, negatively correlated with passive-constructive, active-destructive and passive-destructive. This suggests that the higher the levels of professional identification and active-constructive, the lower the levels of job burnout. Therefore, in order to reduce the job burnout levels of newly recruited nurses, it is necessary to enhance the sense of value and honor of nursing work and pay attention to the response to positive events. It is reported that a strong sense of professional identity can help newly recruited nurses to love nursing work from the heart, feel the sense of meaning and mission brought by the profession, and thus alleviate job burnout (28). Active-constructive response can strengthen the positive behavior of the sharer, which is conducive to the cultivation of personal Professional identification and the construction of a positive atmosphere in the team. Positive psychology believes that positive sharing is an interpersonal emotion regulation strategy that can enhance positive emotions and team belonging, especially when receiving positive feedback, the more positive psychological quality development can be promoted (29). Nursing managers should pay attention to the sharing of positive events of newly recruited nurses, provide an open and relaxed communication environment, reduce the psychological pressure brought by nursing work, and promote the strengthening and dissemination of positive emotions and positive behaviors in the nursing team.

### Mediation effect analysis

This study confirmed that professional identity has a complete mediating effect (72.22%) between passive-constructive responses and job burnout, and a partial mediating effect (59.46%) between passive-destructive responses and job burnout. This result can be deeply interpreted by combining the JD-R model and similar international studies. From the perspective of the JD-R model, professional identity, as a core personal resource, can buffer the pressure caused by job demands (8).

For passive-constructive responses, their “plain recognition” trait, though not directly negative, lacks in-depth empathy for new nurses' positive events and cannot strengthen their perception of professional value, which is consistent with the conclusion of Jiang et al. on ICU nurses that low-quality capitalization support weakens nurses' professional identity (16). Internationally, American scholars Brudner et al. also pointed out that social feedback lacking engagement inhibits the development of individuals' positive psychological qualities, thereby reducing professional identity (29), which explains why passive-constructive responses can only affect job burnout completely through professional identity.

The partial mediating effect of passive-destructive responses stems from the direct blow of this mode to new nurses' work enthusiasm and the indirect erosion of professional identity, and this mechanism is cross-culturally universal. A study on European nurses by Zborowska et al. found that the weakening of professional identity directly exacerbates emotional exhaustion and depersonalization (9), which is consistent with the negative predictive effect of professional identity on job burnout in this study (*B* = −1.51, *P* < 0.001). A study by Wen et al. on nurses in Guangdong also confirmed that negative social interactions directly reduce work engagement and indirectly weaken professional identity (30), verifying the dual-path effect of passive-destructive responses.

### Implications for clinical practice

Based on the results of this study and combined with international nursing management experience, a multi-dimensional intervention system of “positive feedback-professional identity-burnout relief” can be constructed to address the global crisis of new nurse turnover. First, integrate active capitalization response capability training into the nursing training system, which can draw on the “authentic leadership-based positive feedback mechanism” in Canada healthcare institutions—managers strengthen new nurses' perceived professional value by proactively listening to their feedback on positive work events and providing specific, enthusiastic recognition (rather than plain responses) (4). This study also confirmed that such responses can improve professional identity and thus reduce burnout.

Second, targeting the mediating role of professional identity, the occupational transition training program implemented by Hu et al. in the intensive care unit can be promoted and adapted to different countries locally (10). For example, Europe can add professional value narrative modules based on its nursing humanistic tradition, and Southeast Asia can incorporate team identity training under collectivist culture to strengthen new nurses' professional belonging.

In addition, it is necessary to establish a psychological support space across departments and introduce internationally mature positive psychological group counseling techniques to encourage new nurses to regularly share positive events at work (31). A study by Spence Laschinger et al. showed that resource supplementation interventions based on the JD-R model can reduce the incidence of job burnout among new nurses by 22% (8), and this study suggests that incorporating “capitalization responses” into the scope of resource supplementation can further improve the intervention effect.

### Limitations

This study has certain limitations. First, the research objects of this study are only newly recruited nurses in two tertiary hospitals in Wuhan, China. The generalizability of the research results is limited by the regional and hospital-level characteristics of the research sample, and it cannot be extended to nursing groups in other regions of China and other countries for the time being. Subsequent studies need to expand the sample scope and carry out cross-cultural studies to further verify the applicability of the research conclusions. Second, this study adopted a cross-sectional research design, which only uncovers the correlational relationships among the three variables and cannot clarify the causal relationships and dynamic variation laws between them. A prospective longitudinal study is planned for future research.

## Conclusion

For the first time, this study revealed the differentiated mediating mechanism of professional identification between perceived responses to capitalization attempts and job burnout among newly recruited nurses under the dual theoretical framework of Job Demands-Resources Model and positive psychology, expanding the research perspective on influencing factors of new nurses' job burnout. The research findings provide innovative empirical references for the Chinese nursing management field to formulate targeted psychological support strategies, improve new nurses' professional identification, alleviate job burnout and address factors associated with nursing workforce instability.

## Data Availability

The raw data supporting the conclusions of this article will be made available by the authors, without undue reservation.

## References

[1] Xie J Li J Wang S Li L Wang K Duan Y . Job burnout and its influencing factors among newly graduated nurses: a cross-sectional study. J Clin Nurs. (2021) 30:508–17. doi: 10.1111/jocn.15567 33205476

[2] Gong S Li J Tang X Cao X. Associations among professional quality of life dimensions, burnout, nursing practice environment, and turnover intention in newly graduated nurses. Worldviews on evidence-based nursing. (2022) 19:138–48. doi: 10.1111/wvn.12568 35297536

[3] Cañadas-De la Fuente GA Vargas C San Luis C García I Cañadas GR De la Fuente EI. Risk factors and prevalence of burnout syndrome in the nursing profession. Int J Nurs Stud. (2015) 52:240–9. doi: 10.1016/j.ijnurstu.2014.07.001 25062805

[4] Boamah SA Read EA Spence Laschinger HK. Factors influencing new graduate nurse burnout development, job satisfaction and patient care quality: a time-lagged study. J Adv Nurs. (2017) 73:1182–95. doi: 10.1111/jan.13215 27878844

[5] Chao M Shih C-T Hsu S-F. Nurse occupational burnout and patient-rated quality of care: The boundary conditions of emotional intelligence and demographic profiles. Jpn J Nurs Sci. (2016) 3:156–65. doi: 10.1111/jjns.12100 26542752

[6] Jang SJ Wang WX Yang X Tian HG Chen ML. Studying on the factors to influence the job burnout of medical staffs in Shenzhen city. The Chinese Health Service Management. (2020) 37:709–12.

[7] Roberts SJ. Development of a positive professional identity: liberating oneself from the oppressor within. ANS Adv Nurs Sci. (2000) 22:71–82. doi: 10.1097/00012272-200006000-00007 10852670

[8] Spence Laschinger HK Grau AL Finegan J Wilk P. Predictors of new graduate nurses' workplace well-being: testing the job demands-resources model. Health Care Manage Rev. (2012) 37:175–86. doi: 10.1097/HMR.0b013e31822aa456 21799432

[9] Zborowska A Gurowiec PJ Młynarska A Uchmanowicz I. Factors affecting occupational burnout among nurses including job satisfaction, life satisfaction, and life orientation: a cross-sectional study. Psychol Res Behav Manag. (2021) 14:1761–77. doi: 10.2147/PRBM.S325325 34744464 PMC8566003

[10] Hu F Ding XB Zhang RH Li SY Cao R Deng L . A transition programme to enhance ICU new graduate nurses' professional identity and intention to remain employed: a pre- and postevaluation. Nurs Open. (2023) 10:1517–25. doi: 10.1002/nop2.1401 36175954 PMC9912439

[11] Gable SL Reis HT Impett EA Asher ER. What do you do when things go right? The intrapersonal and interpersonal benefits of sharing positive events. J Pers Soc Psychol. (2004) 87:228–45. doi: 10.1037/0022-3514.87.2.228 15301629

[12] Kao FH Kao CC. Why and how the interpersonal stressors influence nurses' intention to stay and job satisfaction: the JD-R model perspective. J Health Organ Manag. (2024) 38:1280–98. doi: 10.1108/JHOM-03-2024-0075 40934215

[13] Zeng D Takada N Hara Y Sugiyama S Ito Y Nihei Y . Impact of intrinsic and extrinsic motivation on work engagement: A cross-sectional study of nurses working in long-term care facilities. Int J Environ Res Public Health. (2022) 19:1284. doi: 10.3390/ijerph19031284 35162307 PMC8834725

[14] Demir M Haynes A Potts SK. My friends are my estate: friendship experiences mediate the relationship between perceived responses to capitalization attempts and happiness. J Happiness Stud. (2017) 18:1161–90. doi: 10.1007/s10902-016-9762-9

[15] Chen MR Jiang YF Yu CH. Correlation between perceived responses to capitalization attempts and work flow of clinical nurses in some class III grade A hospitals of Hangzhou city. CJMN. (2021) 27:383–87.

[16] Jiang QQ Chen YH Diao QX Guo Y. Study on the influence of psychological capital and capitalization support on job burnout in ICU nurses. J Nurs. (2020) 27:74–8.

[17] Smith SM Reis HT. Perceived responses to capitalization attempts are influenced by self-esteem and relationship threat. Pers Relationship. (2012) 19:367–85. doi: 10.1111/j.1475-6811.2011.01367.x

[18] Gustafsson JE Martenson R. Structural equation modeling with AMOS: basic concepts, applications, and programming. Contemporary Psychology APA Review of Books. (2002) 47:478–80. doi: 10.1037/001198

[19] Duan W Yang JJ Fang PP Zhu WJ Zhang Y Li XY. et al. Association between COVID-19 history and postoperative delirium in elderly patient undergoing elective surgery: a prospective, two-center observational cohort study. Anesthesiol Perioper Sci. (2025) 3. doi: 10.1007/s44254-025-00088-4

[20] Zhu W Lou XP Wang ZM. The study of construct validity and reliability of the Maslach burnout inventory-general survey (MBI-GS) for nurses. Chin J Behav Med Sci. (2007) 16:849–51.

[21] Brown R Condor S Mathews A Wade G Williams J. Explaining intergroup differentiation in an industrial organization. J Occup Psychol. (1986) 59:273–86. doi: 10.1111/j.2044-8325.1986.tb00230.x

[22] Xia F Xu L Li G Wang Y Chen G Wang X . Mediating effect of meaning in life in the relationship between empathy for pain and prosocial behavior among nursing students: A multi-center cross-sectional study. SAGE open nursing. (2025) 11:23779608251393309. doi: 10.1177/23779608251393309 41209029 PMC12589795

[23] Li Q Xia F Wang G Chen R Chen G. Effect of mental state on sleep quality in patients receiving maintenance hemodialysis: a multiple mediation model of hope and family function. Medicine. (2024):103:e40503. doi: 10.1097/MD.0000000000040503 39533620 PMC11556987

[24] Wang XF Xia FH & Wang GQ. Mediating effect of anxiety and depression between family function and hope in patients receiving maintenance hemodialysis: a cross-sectional study. BMC psychology. (2023):11:130. doi: 10.1186/s40359-023-01169-4 37098642 PMC10127155

[25] Lay KS Masingboon K. Turnover prevalence and the relationship between transition shock and turnover intention among new nurses: A meta-analysis. Int J Nurs Stud Adv. (2025) 9:100390. doi: 10.1016/j.ijnsa.2025.100390 40842506 PMC12365110

[26] Zang Q Liu LX Yang H. Mediating effect of benefit support on psychological resilienceand death anxiety of NICU nurses. J Bengbu Med Univ. (2022) 47:675–79.

[27] Zhai SQ Dai CC Liu QQ Lu YF Chen CR. Clinical nurses' job embeddedness and its relationship to professional identity, organizational climate, and compassion catigue: a structural equation model. J Nurs Manag. (2025) 2025:3700369. doi: 10.1155/jonm/3700369 40959603 PMC12436005

[28] Wang GQ Fan H Tan LH. Mediating effect of professional identity between feedback-seeking behavior and job burnout among newly recruited nurses. Chin J Ind Med. (2022) 35:533–35.

[29] Brudner EG Fareri DS Shehata SG Delgado MR. Social feedback promotes positive social sharing, trust, and closeness. Emotion. (2023) 23:1536–48. doi: 10.1037/emo0001182 36355668 PMC10169536

[30] Wen RH Luo L Wen ZH Wen XX Yan R Luo YC. Influence of negative emotion and social support on professional identity of nurses in Guangdong Province during COVID-19 pandemic. Chinese occupational medicine. (2020) 47:695–700.

[31] Martins Irvine A Moloney W Jacobs S Anderson NE. Support mechanisms that enable emergency nurses to cope with aggression and violence: Perspectives from New Zealand nurses. Australas Emerg Care. (2024) 27:97–101. doi: 10.1016/j.auec.2023.09.003 37743125

